# Mechanisms of Pulmonary Escape and Dissemination by *Cryptococcus neoformans*

**DOI:** 10.3390/jof4010025

**Published:** 2018-02-17

**Authors:** Steven T. Denham, Jessica C. S. Brown

**Affiliations:** Division of Microbiology and Immunology, Department of Pathology, University of Utah, 15 N Medical Drive, Salt Lake City, UT 84112, USA; steven.denham@utah.edu

**Keywords:** *Cryptococcus*, pulmonary, macrophages, intracellular proliferation, epithelial cells, dissemination, disease progression, capsule, GXM, cell morphology

## Abstract

*Cryptococcus neoformans* is a common environmental saprophyte and human fungal pathogen that primarily causes disease in immunocompromised individuals. Similar to many environmentally acquired human fungal pathogens, *C. neoformans* initiates infection in the lungs. However, the main driver of mortality is invasive cryptococcosis leading to fungal meningitis. After *C. neoformans* gains a foothold in the lungs, a critical early step in invasion is transversal of the respiratory epithelium. In this review, we summarize current knowledge relating to pulmonary escape. We focus on fungal factors that allow *C. neoformans* to disseminate from the lungs via intracellular and extracellular routes.

## 1. Introduction

The lungs present a mucosal barrier that is in constant contact with airborne microbes, including fungi. In fact, it is estimated that every breath contains 1–10 fungal spores [1]. Many human fungal pathogens, such as *Cryptococcus neoformans*, *Aspergilllus* spp., *Coccidiodes immitis*, *Blastomyces dermatitis*, and *Histoplasma capsulatum,* initiate infection via inhalation [2]. The lung mucosa can prevent these fungi from invading deeper tissues, where they can cause more harm and become increasingly difficult to treat [2,3,4]. Thus, it is paramount for the host to maintain proper barrier function and immunosurveillance in order to prevent the invasion of potentially harmful microbes [5]. Respiratory epithelial cells and the tight junctions that bridge them form a physical barrier against microbial invasion [6]. The lung epithelium also plays a constitutive role in regulating microbial load through mechanisms such as secretion of antimicrobial defensins and mucociliary clearance [6]. Both resident professional immune cells and epithelial cells are capable of sensing and coordinating immune responses to microbes [7].

One prevalent human fungal pathogen that begins its infectious lifestyle in the lungs is *Cryptococcus neoformans. C. neoformans* is globally distributed and extremely common in the environment, where it is frequently associated with trees and bird guano [8]. *C. neoformans* and its sibling species, *Cryptococcus gattii*, are the primary cause of fungal meningitis worldwide [4,9]. *C. neoformans* primarily infects individuals with a low CD4^+^ T cell count [4], particularly patients with AIDS; *C. gattii* is more likely to infect immunocompetent individuals [10,11].

Infections begin via inhalation of desiccated *C. neoformans* cells or spores [12,13]. Human exposure to *C. neoformans* is predicted to be near universal, first occurring in early childhood [14]. In immunocompromised patients, *C. neoformans* may be completely cleared by the immune system, or persist asymptomatically for indefinite periods of time within granulomas [15,16]. *C. neoformans* is capable of operating as a facultative intracellular pathogen, and as such control of *C. neoformans* is reliant on robust cell-mediated immunity, characterized broadly by a T-helper 1 (Th1) profile [17]. This includes production of cytokines such as interleukin 12 (IL-12), interferon gamma (IFNγ), tumor necrosis factor alpha (TNFα), and classically activated, or M1, skewing monocytes/macrophages [17]. T-helper 2 (Th2) associated responses, such as production of interleukin 33 (IL-33), (interleukin 13) IL-13, (interleukin 4) IL-4 cytokines, and eosinophilia, are generally considered to be detrimental [17]. Much effort is directed at working out the distinct mechanisms governing immunity to *C. neoformans* and the feasibility of vaccine development [17,18].

In immunocompromised individuals, either persistent or newly acquired *C. neoformans* can proliferate within the lungs and disseminate to basically any organ in the body. *C. neoformans* shows a particular predilection for the CNS, and is able to cross the blood–brain barrier to cause devastating meningitis. Cryptococcal meningitis causes approximately 15% of AIDS deaths annually [4]. Treatment is especially difficult because the central nervous system (CNS) stage of disease is both the presenting illness and the driver of mortality [9].

Although cryptococcal pneumonia does account for a potentially underdiagnosed disease burden [19,20], the most life-threatening presentation is cryptococcal meningitis [4]. Before *C. neoformans* can invade deeper tissues of the host, such as the CNS, it must first escape from the lungs. Additionally, the state of *C. neoformans* exiting the lungs can influence later stages in dissemination, such as transversal of the blood–brain barrier [21]. In this review, we explore processes leading to *C. neoformans*’s escape from the lungs and dissemination throughout the host.

## 2. Routes of Escape

**Figure 1:**
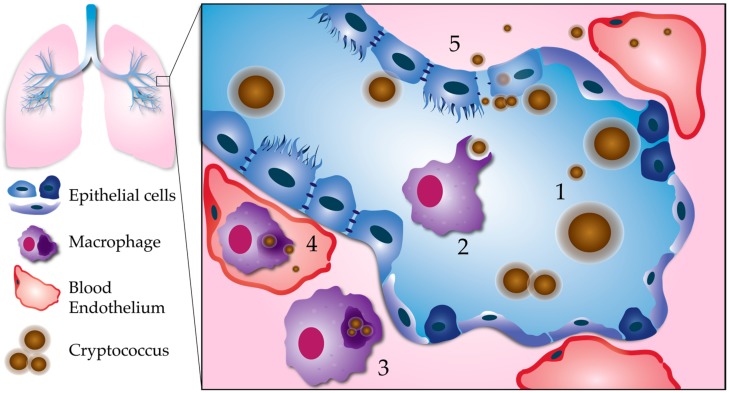
Processes leading to pulmonary escape by *C. neoformans*. (1) Fungal factors influencing pulmonary escape: Proliferation within the lungs results in phenotypic diversity. Cell body, cell wall, and capsule enlargement promote adaptation to host stresses, and evasion of the host immune system. This reservoir of resilient cells may generate smaller cells with a greater propensity for dissemination. (2) Phagocytosis: Smaller, less encapsulated cells are more easily phagocytosed, especially with the aid of opsonization. (3) Intracellular survival: *C. neoformans* is able to survive and replicate within the phagolysosome of host phagocytes, such as macrophages. Macrophages containing *C. neoformans* act as “Trojan horses” to carry fungal cells across the respiratory epithelium. (4) Escape from macrophages: Macrophages can carry *C. neoformans* to distal sites, such as the brain microvasculature. *C. neoformans* can escape from macrophages at unknown stages in dissemination via nonlytic exocytosis, or vomocytosis. Here, we show vomocytosis occurring within an alveolar-associated capillary, but it could potentially take place at multiple sites within the body, including the blood–brain barrier. (5) Extracellular escape: *C. neoformans* also has the potential to escape the lungs independent of macrophages. After adhering to the respiratory epithelium, *C. neoformans* may cross through epithelial cells in a process called transcytosis. Lysis of epithelial cells and/or disruption of epithelial cell tight junctions could allow for *C. neoformans* to cross in between epithelial cells (paracellular crossing). Blood-borne extracellular *C. neoformans* must adapt to alkaline physiological pH (pH 7.3).

There are two main routes by which *C. neoformans* is predicted to escape the lungs and invade deeper tissues (Figure 1). The first is an intracellular route within macrophages or other phagocytes migrating out from the lungs, known as the “Trojan horse” mechanism [21,22]. The second is the escape of extracellular *C. neoformans* cells. This could occur via transcytosis, when fungal cells pass directly through respiratory epithelial cells [23]. It could also occur after tissue damage allows for the passage of *C. neoformans* cells between epithelial cells, a process we will refer to as paracellular crossing [23].

### 2.1. Intracellular Escape

Among the first professional immune cells that *C. neoformans* encounters are resident alveolar macrophages and dendritic cells [24]. Interactions of *C. neoformans* with these patrolling phagocytic cells is key in determining the outcome of infection. Properly activated macrophages can be proficient at ingesting and destroying *C. neoformans* cells [25,26]. However, *C. neoformans* has a suite of traits that allow it to survive phagocytosis by macrophages and even proliferate within and escape from the phagolysosome. Additionally, macrophages could facilitate *C. neoformans* dissemination from the lungs and even transversal of the blood–brain barrier [22,27]. In order to use macrophages as a route of escape, *C. neoformans* must accomplish three important tasks: (1) entering the macrophage (phagocytosis), (2) surviving within the macrophage, and (3) escaping from the macrophage once outside the lung.

#### 2.1.1. Phagocytosis

The first point, entrance into a macrophage or other phagocyte, seems straightforward: phagocytosis is a primary task for macrophages. However, *C. neoformans* is able to evade phagocytosis, particularly when it has not been opsonized by antibodies or complement [28]. Since many cryptococcosis patients are immunocompromised, the host antibody response could well be delayed, allowing for a period of fungal cell growth and/or formation of Titan cells (see Section 3.1) in the lungs. Experiments in zebrafish found that most *C. neoformans* cells are found outside macrophages at 2 h post-inoculation (hpi) but that almost >50% of fungal cells were found within macrophages at 24 hpi [29].

Phagocytosis evasion, sometimes called anti-phagocytosis, is heavily influenced by *C. neoformans*’s polysaccharide capsule, which cloaks the highly immunogenic cell wall. By mass, the capsule is comprised of ~90% glucuronoxylomannan (GXM), ~9–10% galactoxylomannan (GXMGal), and ~1% mannoproteins [30,31,32]. Unopsonized cells are rarely phagocytosed [28]: ~1% of RAW264.1 cells will associate with a cryptococcal cell in in vitro experiments [33]. When opsonized, that number increases to >20% [34]. Fungal cells without cell surface capsule are rapidly phagocytosed and destroyed [28]. In vivo imaging found that *C. neoformans* cells that were not phagocytosed 24 hpi had larger capsules than those that were phagocytosed [29].

GXM that is unattached to the fungal cell surface, or exo-GXM, is also known to accumulate in macrophages [35]. Since GXM inhibits a variety of phagocyte functions [36], accumulation of exo-GXM could serve as a method for suppressing macrophage activity during cryptococcal disease.

Phagocytosis evasion also functions independently from the cryptococcal cell capsule [33,37,38]. App1, a secreted protein involved in phagocytosis evasion by opsonized cells [38] binds to complement receptors CR2 and CR3, which can also serve as opsonins [39]. The molecular mechanisms underlying phagocytosis evasion by unopsonized cells are poorly understood, but likely involve multilayer transcriptional networks controlled by the master regulator Gat201 [33,37].

#### 2.1.2. Intracellular Survival

Once *C. neoformans* has been phagocytosed, it must survive and replicate intracellularly. An important trait is *C. neoformans*’s ability to survive low pH environments [40]. This ability likely results from one of *C. neoformans*’s environmental niches: bird guano is acidic and rich in nitrogen. *C. neoformans* replicates more rapidly within tissue culture macrophages than in tissue culture medium itself [41], and is capable of lysing macrophages from within [24]. Phagolysosomes containing *C. neoformans* cells show an initial pH decrease to pH 4.7, which then increases to pH 5.3 [42]. A second recent report also indicates that the phagolysosome is not fully acidified [43], indicating some disruption to the phagolysosome. However, it appears to be more acidic than commonly seen when macrophages are infected with other pathogens that prevent full maturation of the phagolysosome, such as *Legionella pneumophila* [44]. This strategy of surviving low pH, rather than completely blocking acidification of the phagolysosome, could help maintain *C. neoformans*’s access to iron, as iron is bound to transferrin at neutral pH and cannot be utilized by invading microbes [42,45]. Additional evidence supporting the importance of *C. neoformans’s* affinity for low pH environments comes in the form of a secreted phospholipid modifying enzyme, phospholipase B1 (Plb1). Plb1 can be anchored to the cell wall or secreted into the extracellular milieu, and has an optimal activity in the acidic pH ranges of the phagosome [46,47,48]. Plb1 promotes survival in macrophages, and is required for efficient dissemination from the lungs [49,50].

Evaluation of phagosomal markers indicates that *C. neoformans*-containing phagosomes do undergo initial maturation. The early endosomal and phagosomal marker Rab5, which recruits later phagosome and phagolysosome proteins, is indeed found on *C. neoformans*-containing phagosomes. Rab5 and Rab11 rapidly disappear (<15 min) from these compartments in *C. neoformans*-infected cells compared to cells containing heat killed *C. neoformans*, in which Rab5 and Rab11 associate with the phagosome for longer than 120 min [43]. *C. neoformans*-containing phagosomes show increased permeabilization early in the maturation process [51,52,53].

The cell surface capsule is also necessary for cryptococcal cell survival following phagocytosis, as acapsular cells cannot replicate inside macrophages [24]. Some of this could be due to reactive oxygen and nitrogen species released by the host, as capsule protects against these insults [54]. However, the reactive oxygen burst produced by macrophages is weaker than the neutrophil burst [55]. Powerful microscopy work in the zebrafish model of cryptococcosis actually found that, following phagocytosis by macrophages, the size of the cell surface capsule decreases [29]. In addition, *C. neoformans* cells replicating within the phagolysosome release GXM that accumulates within cytoplasmic vesicles [51]. Since our work suggests that cell surface capsule size and unattached, or exo-GXM, is inversely correlated, exo-GXM could also play a role in *C. neoformans* survival within macrophages [56].

Cryptococcal survival within macrophages also depends on the macrophage’s activation state. *C. neoformans* can induce a Th2 immune response in the lungs [57]. This response is correlated with disease progression: a Th2 response, with induction of type 2 cytokines, correlates with a pulmonary infection [58,59]. In contrast, when a Th1 response is encouraged by infecting mice with a *C. neoformans* strain expressing the type 1 cytokine IFNγ, mice are protected against further infection and macrophages show an M1 polarization pattern [60]. Since *C. neoformans* cells are less efficiently killed by M2 than M1 polarized macrophages [25,26], controlling macrophage activation state could be a powerful strategy to promote fungal survival. Evidence suggests that a number of fungal factors, including urease [58,59] and the Hsp70 family protein Ssa1 [61] promote M2 polarization in macrophages.

#### 2.1.3. Escape from Macrophages

*C. neoformans* can escape from macrophages through a nonlytic process called vomocytosis [62]. This escape ability is critical for the Trojan horse hypothesis of cryptococcal dissemination.

After phagocytosis, *C. neoformans* is engulfed in the phagosome. To escape, *C. neoformans* first permeabilizes the phagosome by an unknown mechanism [51,52]. Next, actin rapidly and transiently polymerizes in a cage-like structure around the phagosome [52]. Finally, the *C. neoformans* cell exits the macrophage. The actin nucleator proteins Arp2/3 are necessary for the rapid formation of the actin cage-like structures [52]. These cages might be a post-phagosome permeabilization attempt by the macrophage to inhibit *C. neoformans* cells’ escape, as inhibiting actin polymerization increases vomocytosis. Vomocytosis is also regulated by the mitogen-activated protein kinase (MAP kinase), extracellular-signal-regulated kinase 5 (ERK5), and pharmacological inhibition of ERK5 increases vomocytosis [63]. As vomocytosis increases, dissemination decreases, suggesting that there is a balance between too rapid an escape and spread to distal sites within the host [63].

*C. neoformans* cells can also transfer laterally between macrophages, lyse macrophages, and escape when a macrophage divides or fuses with another macrophage. These processes are all poorly understood, but are reviewed in [64,65].

### 2.2. Extracellular Escape

Another potential, but not mutually exclusive route of escape for *C. neoformans*, is the transversal of fungal cells across the lung mucosa and into the blood-stream or lymphatics. To accomplish this, *C. neoformans* cells need to subvert the barrier function of epithelial cells separating them from the pulmonary interstitial space and vasculature. This can be achieved through either tissue damage and paracellular crossing, or transcytosis across respiratory epithelial cells. In vitro characterization of *C. neoformans* with the A549 human alveolar cell line has demonstrated that *C. neoformans* can adhere to, enter, and also damage lung epithelial cells [23,66,67,68,69,70].

Multiple factors have been implicated in the ability of *C. neoformans* to adhere to respiratory epithelial cells, which requires interacting surface features of both *C. neoformans* and the epithelial cells. On the fungal side, adherence is likely mediated by capsule GXM, which binds to CD14 on epithelial cells [67,68]. This interaction can also result in the internalization of *C. neoformans* and epithelial cell lysis [67]. Live *C. neoformans* cells display increased adherence over dead *C. neoformans* cells, and only live cells induce epithelial cell lysis, indicating that *C. neoformans* plays an active role in entry [67,70]. The mannoprotein MP84 is also implicated in playing a role in the adherence of poorly encapsulated *C. neoformans* cells [69]. MP84 is a component of the capsule, but purified MP84 cannot competitively inhibit adherence of encapsulated *C. neoformans*, suggesting that GXM and MP84 engage epithelial cells via separate mechanisms [69].

In addition to its important role in interacting with macrophages, phospholipase B1 (Plb1) is implicated in promoting adhesion to A549 lung epithelial cells [70]. Although the precise mechanism is not yet determined, the phospholipase B function, and not the lysophospholipase, or lysophospholipase transacylase functions of the multifunctional Plb1 enzyme is suggested to be most important for promoting adherence [70].

Little is known about how *C. neoformans* might enter and transverse respiratory epithelial cells after adhesion, a process known as transcytosis [66,67]. Transcytosis also occurs when *C. neoformans* cells cross the blood–brain barrier [21]. However, the frequency and relevance of respiratory epithelial cell transcytosis during infection is unclear. If transcytosis of the lung epithelium does occur, one host factor that could play a role is annexin A2 (ANXA2). ANXA2 is a membrane binding protein that facilitates membrane fusion, and is thus important for vesicle trafficking and other related processes. ANXA2 is critical for both transcytosis of the brain endothelium and non-lytic exocytosis from macrophages [71,72,73]. Thus, it is possible that ANXA2 also mediates transcytosis of the lung epithelium. Another candidate protein that could facilitate transcytosis is EphA2. This tyrosine kinase receptor is necessary for *C. neoformans* cells’ ability to cross the blood–brain barrier [74] and the *EPHA2* gene is expressed in lung tissue [75].

Respiratory epithelial cells are also capable of recognizing potential pathogens and initiating innate immune responses through the early production of cytokines and chemokines [7]. *C. neoformans* interactions with epithelial cells in vitro results in the secretion of the chemokines IL-8 and CXCL1, which recruit monocyte and neutrophils, and could provide an early signal for host-defense [7,68,76,77]. However, a recent in vivo study of pulmonary cryptococcosis shows that *C. neoformans* can induce potent IL-33 production from the lung epithelia, promoting non-protective Th2 responses [78]. Additionally, IL-33 production leads to a decrease in tight junction E-cadherin, which could disrupt intracellular barrier function and provide a potential paracellular route of escape for *C. neoformans* [78]. More work is required to determine the mechanisms by which inflammatory signals from the lung epithelium mediate containment or escape of pulmonary *C. neoformans*.

## 3. Examples of Fungal Factors Influencing Pulmonary Escape

In the following sections, we highlight selected fungal factors that are particularly important for disease progression and escaping the lungs.

### 3.1. Cell Morphology

After inhalation, *C. neoformans* is capable of dramatically enlarging its size via two main mechanisms that ward against early clearance by the immune system: (1) *C. neoformans* produces a polysaccharide surface capsule that can double the 5–10 μm diameter of a typical haploid yeast cell grown in vitro [32]. The production of surface capsule is critical for all stages of virulence, as acapsular variants are quickly cleared after murine inoculation [79,80] and are rarely seen in human patients [81]. The mechanisms governing capsule synthesis have been extensively reviewed elsewhere [30,31,32]; and (2) *C. neoformans* increases its cell body size, producing enormous Titan cells that measure up to 100 μm in diameter in extreme cases [82,83,84,85,86].

Titan cell formation is regulated by multiple environmental signals, including stimulation by mating pheromone [85] and host phospholipids [87], and signal transduction through G-coupled protein receptors [88]. The G-protein coupled receptors Gpr4 and Gpr5 are especially important, as deletion of both *gpr*4 and *gpr*5, (*gpr4*Δ/*gpr5*Δ) results in a severe deficiency in Titan cell formation [88,89]. The protein kinase A pathway also plays a role in regulating Titan cell formation through the pleiotropic, pH responsive transcription factor Rim101, since *rim101*Δ cells are severely deficient for Titan cell formation [90,91]. Titan cells exhibit thickened cell walls, and a denser capsule structure [82,83,84,85,86], which may contribute to their increased resistance to oxidative and nitrosative stresses [85,92], therapeutic antifungals [92], and phagocytosis [92,93]. Some of the anti-phagocytic effect is due to the sheer size of Titan cells. However, the presence of Titan cells can also protect smaller cells from phagocytosis, so there are likely other mechanisms at work [93]. 

Titan cells are polyploid, which is likely a mechanism to support their increased size [84,85,92]. Notably, Titan cells bud smaller haploid and aneuploid daughter cells, which can inherit some stress resistance from their mothers, demonstrating how Titan cells could support population level adaptation to host stresses [84,92].

Titan cells are implicated in establishing infection and progressing disease within the lungs. The Titan cell-deficient *gpr4*Δ/*gpr5*Δ mutant displays decreased virulence, reduced lung proliferation, and greatly reduced dissemination to the spleen and brain of infected mice [88,89]. Increased Titan cell formation is associated with detrimental lung eopsinohilia and general Th2 immune skewing [89]. In addition, C57BL/6 mice, which are prone to respond to *C. neoformans* with a strong Th2 profile, have more Titan cells in their lungs than CD1 mice, which trend towards Th1 immune responses [94]. The precise casual relationships behind these associations will require additional studies, although greater Th2 activation could be related to increased chitin content in Titan cell walls [95].

Median cell and capsule sizes vary by infected tissue [56,96,97]. Titan cells are rarely observed outside of the lungs, and cells found in the brain typically display smaller median cell diameter and capsule thickness than those in the lungs, suggesting that cell size could influence proclivity for dissemination [56,96,98]. We recently reported that median cell and capsule size decrease proportionally over time in the lungs of intranasally inoculated C57BL/6 mice, from around 30 µm early after inoculation, to approximately 10 µm late in infection [56]. Fungal proliferation in the lungs and the emergence of small cells coincided with extrapulmonary dissemination. Additionally, a mutant strain (*liv7*Δ) that exhibited a slower transition to smaller median cell size also disseminated at a reduced rate [56]. Importantly, both wild-type and *liv7*Δ cells showed equivalent lung fungal burden and cell size early after inoculation, indicating that the differences in dissemination were more likely due to altered disease progression than disease initiation [56].

As mentioned previously, phospholipaseB1 (Plb1) deficiency results in increased uptake by macrophages, reduced intracellular proliferation, and reduced dissemination [49,50]. Interestingly, *C. neoformans* cells lacking Plb1 (*plb1*Δ) form Titan cells at a greater rate than wild-type cells in vivo. *plb1*Δ cells even undergo Titan cell formation after phagocytosis by macrophages, although the latter phenomenon has not been directly observed in vivo [49]. These observations could contribute to the *plb1*Δ mutant’s poorer ability to disseminate from the lungs [49,50].

Dynamic changes in cell and capsule size are likely important features of establishing and progressing infection within the lungs. Robust escape from the lungs possibly involves balancing the proportion of larger cells, which provide protection and resiliency in the lungs, with smaller cells more capable of dissemination.

### 3.2. Age

The aging of *C. neoformans* cells within the lungs is a source of phenotypic variation that potentially enhances extrapulmonary dissemination [99]. *C. neoformans* aging can be categorized as chronological aging or replicative aging. Chronological age refers to the cumulative lifespan of a cell. Replicative age refers to the number of times a cell has divided. Similar to Titan cells, *C. neoformans* cells of increased replicative age are more resistant to clearance by the host, and thus may serve as a reservoir for fungal persistence, although older *C. neoformans* likely disseminate at a greater frequency than Titans [99]. Unlike the model yeast *Saccharomyces cerevisiae*, the *C. neoformans* cell wall strengthens with replicative age, making them more resistant to a variety of stresses, including oxidative stress and therapeutic antifungals [100,101,102]. Additionally, replicative aging results in a proportional increase in both cell size and capsule [101,102], consistent with cell cycle regulation of capsule growth [103]. The increase in size also increases resistance to phagocytosis by macrophages [101,102].

Recent developments in the field of *C. neoformans* aging have implicated the conserved histone deacetylase Sir2 as a regulator of replicative life-span (RLS) [104]. Deletion of the *sir2* gene (*sir2*∆) results in pleiotropic changes to the transcriptome and shortens median RLS by 33%. The *sir2*∆ mutant is hypovirulent in the intranasal, but not the intravenous murine infection model. Notably, mice still succumb to intranasal infection with the *sir2*∆ mutant, but median time to death is considerably delayed, suggesting that Sir2 may be an important disease progression factor influencing dissemination [104].

### 3.3. Melanin

In *C. neoformans*, melanin pigment is exported to the cell wall [105], where it provides protection against ultraviolet radiation and oxidative damage [106,107]. Melanin synthesis requires the enzyme laccase, and two adjacent laccase genes (*lac1* and *lac2*) can be found within the *C. neoformans* genome [108]. Transcription of *lac2* is significantly lower than *lac1*, and as such the greatest defects in melanization are seen when either *lac1* or both *lac1* and *lac2* are disrupted [108].

Multiple studies have suggested that melanin is more important for dissemination and CNS infection than pulmonary infectivity [33,109,110]. Laccase deficient strains are hypovirulent, causing delayed death in intranasally inoculated animals [110], despite wild-type lung infectivity [109]. Reduced extrapulmonary fungal burden is observed after intranasal, but not intravenous inoculation with laccase deficient strains [109]. These data suggest that melanin plays an important role in dissemination from the lungs. Laccase activity of *C. neoformans* isolates from human patients correlate with high in vitro uptake by macrophages, increased in vivo and ex vivo survival in cerebrospinal fluid [111]. Thus, laccase could influence uptake and survival within macrophages, influencing intracellular dissemination.

A systematic gene deletion approach to identify *C. neoformans* genes involved in lung infectivity also did not identify *lac1* or *lac2* as being critical for early growth in the lungs [33]. In some cases, however, genes that reduced melanization also reduced lung infectivity. This leaves the possibility that melanin could play a role in lung infectivity in collaboration with redundant pathways [33].

### 3.4. Phosphate Acquisition

Phosphate is critical for numerous cellular functions. In *C. neoformans*, one particular area of interest is phosphate incorporation into inositol pyrophosphates. These metabolites have pleiotropic roles in cell physiology, including regulation of carbon source utilization and virulence factors [112,113,114]. Recent work points to phosphate acquisition playing a critical role in extrapulmonary dissemination [115]. Loss of Pho4 (*pho4*Δ), a transcription factor that regulates genes related to phosphate acquisition, results in sensitivity to both phosphate deprivation and alkaline pH [115,116]. In a murine inhalation model, the *pho4*Δ mutant exhibits a modest defect in pulmonary proliferation, a much more significant defect in dissemination to the brain, and remains below the limit of detection in the blood-stream [115]. Furthermore, proliferation at physiological pH (pH 7.3), in serum, and in peripheral blood monocytes co-culture is markedly reduced. These observations indicate that phosphate acquisition and alkaline pH stress tolerance is important for escaping the lungs via an extracellular or intracellular route [115].

The Rim101 pathway is also critical for responding to alkaline pH [91,117], but Pho4 and Rim101 seem to activate different gene networks [115,116,117]. Thus, Rim101 and Pho4 may respond to alkaline pH independently, with distinct effects on virulence [90,115]. While the *pho4*Δ mutant is hypovirulent, it still produces wild-type levels of capsule and melanin in vitro [115]. In contrast, the *rim101*Δ mutant displays a severe capsule attachment defect [117], but also a thickened cell wall that induces hyper-inflammatory pathology in the lungs [90,118].

### 3.5. Sphingolipids

Plasma membrane sphingolipids are involved in various cellular functions including signaling, lipid raft architecture, and general membrane stability [119]. Sphingolipids are antigenic, and can influence uptake of *C. neoformans* by macrophages [120,121], intracellular proliferation [122], and overall virulence [123].

A prominent example of a fungal sphingolipid regulating *C. neoformans* dissemination is the plasma membrane glycosphingolipid glucosylceramide (GlcCer). Glucosylceramide synthase, encoded by the *gcs1* gene, is required for synthesis of GlcCer. The *gcs1*Δ mutant cannot grow at physiological pH, but can replicate within the acidic phagolysosome of macrophages, and is contained within lung granulomas upon intranasal inoculation of mice [121,124]. 

The inability of the *gcs1*Δ mutant to escape the lungs is likely due to important structural features of GlcCer itself. For instance, the *smt1* gene encodes a sphingolipid C9 methyltransferase that is required for methylation of the GlcCer sphingosine backbone, a modification that distinguishes fungal GlcCer from mammalian GlcCer [125]. The *smt1*Δ mutant maintains a constant but contained presence in the lungs. Accumulation of de-methylated GlcCer and altered membrane organization of the *smt1*Δ mutant perhaps alter its ability to adapt to the host environment and progress disease [125]. In addition to the GlcCer methylation, the saturation state of position 8 of the GlcCer sphingosine backbone influences virulence. Deletion of the *sld8* gene (*sld8*Δ), which is required for desaturation of GlcCer at sphingosine backbone position C8, results in the accumulation of saturated GlcCer [126]. Similar to the *smt1*Δ mutant, the *sld8*Δ mutant displays a delayed growth defect in the lungs. The *sld8*Δ mutant also shows no detectable dissemination to the brain, despite canonical virulence factors remaining intact [126]. Increased membrane permeability of the *sld8*Δ mutant renders it more sensitive to stresses, and in contrast to the *gcs1*Δ mutant, reduces intracellular proliferation rates [126].

While GlcCer appears to be overall more important for extracellular growth in the host at alkaline pH, inositol phosphosphingolipid biology seems to be critical for regulating intracellular growth at acidic pH [122,127]. In *C. neoformans*, the *isc1* gene encodes inositol phosphosphingolipid-phospholipase C1, an enzyme that breaks down inositol sphingolipids. The *isc1*Δ mutant grows poorly at acidic pH, likely due to improper oligomerization of the Pma1 proton pump at the plasma membrane [127]. The *isc1*Δ mutant is slightly attenuated for lung proliferation, but shows very poor dissemination to the brain [122]. Notably, depletion of macrophages rescues neuro-dissemination of the *isc1*Δ mutant. This indicates that *isc1* is important for surviving interactions with macrophages, but, in their absence, dissemination of extracellular *isc1*Δ mutant cells remains possible [122]. Sphingolipid biology highlights the importance of the facultative intracellular lifestyle in *C. neoformans* virulence: disrupting sphingolipid biology to reduce either intracellular or extracellular growth severely hampers extrapulmonary dissemination.

## 4. Conclusions

*C. neoformans* cells’ escape from the lungs is a critical transition from contained (pulmonary) cryptococcosis to advanced, disseminated disease. In the establishment disease phase, fungal cells must persist and replicate in the lungs. Mutants deficient in this stage will exhibit a complete or almost complete attenuation of disease. In contrast, mutants deficient in later stages, such as dissemination, will still cause pulmonary disease, and thus will have more subtle phenotypes. This framework was outlined for infections caused by the filamentous fungus *Aspergillus fumigatus* [128], and is broadly applicable to additional fungal infections. Since cryptococcosis has distinct pulmonary and extra-pulmonary phases, identifying mutants deficient in different phases provides key insights into disease.

In this review, we emphasized fungal factors that influence escape from the lungs. As with any stage of pathogenesis, however, there is a balance between pathogen and host that determines the outcome of infection. In the case of *C. neoformans*, significantly weak or strong immune responses can result in disease [129]. The lungs are the initial site of infection, and the lung mucosa provides the first barrier against invasion, so the outcome of this interaction is critical in determining how immune responses develop and disease progresses [5].

## 5. Future Directions

Both direct epithelial barrier transversal of extracellular *C. neoformans* cells and “Trojan horse”-mediated transversal are possible mechanisms for escaping the lungs. However, the relative frequency of extracellular and intracellular dissemination from the lungs is unknown, and most studies characterizing extracellular crossing were performed in vitro. Both the blood and lymph present possible routes for dissemination. Detecting intracellular and extracellular *C. neoformans* in blood and lymph at different stages of infection could provide a clearer picture of the state of *C. neoformans* exiting the lungs. In the case of the Trojan horse model, it is also important to characterize the host cells carrying *C. neoformans*. Which cell-subtypes are acting as Trojan horses, and what are their migratory patterns from the lungs?

Mice are the most widely used animal model to study disseminated cryptococcosis, and have been of great benefit to the field. However, there are key differences between cryptococcosis in mice and humans that are likely to influence our understanding of pulmonary escape moving forward. Many strains of ostensibly immunocompetent (albeit inbred) laboratory mice succumb to lethal infection with *C. neoformans*, especially when using highly virulent members of the H99 lineage [130]. This is in contrast to most cases of disseminated cryptococcosis in human patients, where there is usually a clear underlying immunodeficiency. In the case of *C. neoformans*, it is most commonly low CD4^+^ T cell count due to HIV/AIDS [4]. This is important because the immune status of the host has broad impacts on pathogenesis, and pulmonary escape could occur differently in immunosuppressed vs. immunocompetent hosts. For example, reconstitution of B cells protects T and B cell deficient Rag1^−/−^ mice against CNS dissemination, but not pulmonary fungal proliferation [131]. This is likely an important observation for human patients, such as T cell deficient HIV/AIDS patients, and even HIV negative patients with various B cell deficiencies [132]. Indeed, HIV negative cryptococcosis patients have fewer B cells that uninfected control patients [132]. Immunodeficiencies can also lead to imbalances that cause normally protective responses to overcompensate and lead to inflammatory mediated damage. Despite reduced lung eosinophil recruitment, Th2 lymphocyte deficient STAT6^−/−^ mice reach endpoints more quickly than wild-type C57BL6/J mice, likely due to over compensatory neutrophil recruitment and excessive pulmonary inflammation [133]. Expanding the repertoire of cryptococcosis in immunodeficient mouse models is likely a powerful way to draw better parallels with the human lung environment leading to pulmonary escape.

Another feature of mouse models to consider is that commonly used strains of C. *neoformans* are able to progress directly from acute pulmonary infection to disseminated disease in laboratory mice, whereas, in humans, there is evidence supporting reactivation of persistent, asymptomatic *C. neoformans* in the lungs [16]. In contrast to mice, immunocompetent laboratory rats inoculated with *C. neoformans* maintain a persistent fungal burden for up to six months [15]. Steroid based immunosuppression leads *C. neoformans* proliferation and dissemination [15]. However, the genetic tools available for rats are lacking in comparison to mice. A reproducible mouse model of *C. neoformans* persistence, and reactivation post-immunosuppression would be of great benefit to the field. Such a model could potentially inform investigators of any differences in pulmonary escape following persistent versus acute infection.

## References

[1] Fröhlich-Nowoisky J., Pickersgill D.A., Després V.R., Pöschl U. (2009). High diversity of fungi in air particulate matter. Proc. Natl. Acad. Sci. USA.

[2] Brown G.D., Denning D.W., Gow N.A.R., Levitz S.M., Netea M.G., White T.C. (2012). Hidden killers: Human fungal infections. Sci. Transl. Med..

[3] Lortholary O., Poizat G., Zeller V., Neuville S., Boibieux A., Alvarez M., Dellamonica P., Botterel F., Dromer F., Chêne G. (2006). Long-term outcome of AIDS-associated cryptococcosis in the era of combination antiretroviral therapy. AIDS.

[4] Rajasingham R., Smith R.M., Park B.J., Jarvis J.N., Govender N.P., Chiller T.M., Denning D.W., Loyse A., Boulware D.R. (2017). Global burden of disease of HIV-associated cryptococcal meningitis: An updated analysis. Lancet Infect. Dis..

[5] Wiesner D.L., Klein B.S. (2017). Lung epithelium: Barrier immunity to inhaled fungi and driver of fungal-associated allergic asthma. Curr. Opin. Microbiol..

[6] Whitsett J.A., Alenghat T. (2014). Respiratory epithelial cells orchestrate pulmonary innate immunity. Nat. Immunol..

[7] Weitnauer M., Mijošek V., Dalpke A.H. (2015). Control of local immunity by airway epithelial cells. Mucosal Immunol..

[8] Mitchell T.G., Castaneda E., Nielsen K., Wanke B., Lazera M.S. (2011). Environmental niches for *Cryptococcus neoformans* and *Cryptococcus gattii*. Cryptococcus: From Human Pathogen to Model Yeast.

[9] Armstrong-James D., Meintjes G., Brown G.D. (2014). A neglected epidemic: Fungal infections in HIV/AIDs. Trends Microbiol..

[10] Kwon-Chung K.J., Fraser J.A., Doering T.L., Wang Z., Janbon G., Idnurm A., Bahn Y.-S. (2014). *Cryptococcus neoformans* and *Cryptococcus gattii*, the etiologic agents of cryptococcosis. Cold Spring Harb. Perspect. Med..

[11] Byrnes E.J., Li W., Lewit Y., Ma H., Voelz K., Ren P., Carter D.A., Chaturvedi V., Bildfell R.J., May R.C. (2010). Emergence and pathogenicity of highly virulent *Cryptococcus gattii* genotypes in the northwest united states. PLoS Pathog..

[12] May R.C., Stone N.R.H., Wiesner D.L., Bicanic T., Nielsen K. (2015). *Cryptococcus*: From environmental saprophyte to global pathogen. Nat. Rev. Microbiol..

[13] Ballou E.R., Johnston S.A. (2017). The cause and effect of *Cryptococcus* interactions with the host. Curr. Opin. Microbiol..

[14] Goldman D.L., Khine H., Abadi J., Lindenberg D.J., Pirofski L.-A., Niang R., Casadevall A. (2001). Serologic evidence for *Cryptococcus neoformans* infection in early childhood. Pediatrics.

[15] Goldman D.L., Lee S.C., Mednick A.J., Montella L., Casadevall A. (2000). Persistent *Cryptococcus neoformans* pulmonary infection in the rat is associated with intracellular parasitism, decreased inducible nitric oxide synthase expression, and altered antibody responsiveness to cryptococcal polysaccharide. Infect. Immun..

[16] Garcia-Hermoso D., Janbon G., Dromer F. (1999). Epidemiological evidence for dormant *Cryptococcus neoformans* infection. J. Clin. Microbiol..

[17] Gibson J.F., Johnston S.A. (2015). Immunity to *Cryptococcus neoformans* and *C. gattii* during cryptococcosis. Fungal Genet. Biol..

[18] Caballero Van Dyke M.C., Wormley F.L. (2017). A call to arms: Quest for a cryptococcal vaccine. Trends Microbiol..

[19] Chang W.-C., Tzao C., Hsu H.-H., Lee S.-C., Huang K.-L., Tung H.-J., Chen C.-Y. (2006). Pulmonary cryptococcosis: Comparison of clinical and radiographic characteristics in immunocompetent and immunocompromised patients. Chest.

[20] Shirley R.M., Baddley J.W. (2009). Cryptococcal lung disease. Curr. Opin. Pulm. Med..

[21] Tseng H.-K., Huang T.-Y., Wu A.Y.-J., Chen H.-H., Liu C.-P., Jong A. (2015). How *Cryptococcus* interacts with the blood–brain barrier. Future Microbiol..

[22] Charlier C., Nielsen K., Daou S., Brigitte M., Chretien F., Dromer F. (2009). Evidence of a role for monocytes in dissemination and brain invasion by *Cryptococcus neoformans*. Infect. Immun..

[23] Taylor-Smith L. (2017). *Cryptococcus*-epithelial interactions. J. Fungi.

[24] Feldmesser M., Kress Y., Novikoff P., Casadevall A. (2000). *Cryptococcus neoformans* is a facultative intracellular pathogen in murine pulmonary infection. Infect. Immun..

[25] Müller U., Stenzel W., Köhler G., Werner C., Polte T., Hansen G., Schütze N., Straubinger R.K., Blessing M., McKenzie A.N.J. (2007). IL-13 induces disease-promoting type 2 cytokines, alternatively activated macrophages and allergic inflammation during pulmonary infection of mice with *Cryptococcus neoformans*. J. Immunol..

[26] Arora S., Olszewski M.A., Tsang T.M., McDonald R.A., Toews G.B., Huffnagle G.B. (2011). Effect of cytokine interplay on macrophage polarization during chronic pulmonary infection with *Cryptococcus neoformans*. Infect. Immun..

[27] Santiago-Tirado F.H., Onken M.D., Cooper J.A., Klein R.S., Doering T.L. (2017). Trojan horse transit contributes to blood–brain barrier crossing of a eukaryotic pathogen. mBio.

[28] Bolaños B., Mitchell T.G. (1989). Phagocytosis and killing of *Cryptococcus neoformans* by rat alveolar macrophages in the absence of serum. J. Leukocyte Biol..

[29] Bojarczuk A., Miller K.A., Hotham R., Lewis A., Ogryzko N.V., Kamuyango A.A., Frost H., Gibson R.H., Stillman E., May R.C. (2016). *Cryptococcus neoformans* intracellular proliferation and capsule size determines early macrophage control of infection. Sci. Rep..

[30] Zaragoza O., Rodrigues M.L., De Jesus M., Frases S., Dadachova E., Casadevall A. (2009). The capsule of the fungal pathogen *Cryptococcus neoformans*. Adv. Appl. Microbiol..

[31] Doering T.L. (2009). How sweet it is! Cell wall biogenesis and polysaccharide capsule formation in *Cryptococcus neoformans*. Ann. Rev. Microbiol..

[32] O’Meara T.R., Alspaugh J.A. (2012). The *Cryptococcus neoformans* capsule: A sword and a shield. Clin. Microbiol. Rev..

[33] Liu O.W., Chun C.D., Chow E.D., Chen C., Madhani H.D., Noble S.M. (2008). Systematic genetic analysis of virulence in the human fungal pathogen *Cryptococcus neoformans*. Cell.

[34] Brown J.C.S., Madhani H.D. (2012). Approaching the functional annotation of fungal virulence factors using cross-species genetic interaction profiling. PLoS Genet..

[35] Chang Z.L., Netski D., Thorkildson P., Kozel T.R. (2006). Binding and internalization of glucuronoxylomannan, the major capsular polysaccharide of *Cryptococcus neoformans*, by murine peritoneal macrophages. Infect. Immun..

[36] Vecchiarelli A., Pericolini E., Gabrielli E., Kenno S., Perito S., Cenci E., Monari C. (2013). Elucidating the immunological function of the *Cryptococcus neoformans* capsule. Future Microbiol..

[37] Chun C.D., Brown J.C.S., Madhani H.D. (2011). A major role for capsule-independent phagocytosis-inhibitory mechanisms in mammalian infection by *Cryptococcus neoformans*. Cell Host Microbe.

[38] Luberto C., Martinez-Mariño B., Taraskiewicz D., Bolaños B., Chitano P., Toffaletti D.L., Cox G.M., Perfect J.R., Hannun Y.A., Balish E. (2003). Identification of app1 as a regulator of phagocytosis and virulence of *Cryptococcus neoformans*. J. Clin. Investig..

[39] Stano P., Williams V., Villani M., Cymbalyuk E.S., Qureshi A., Huang Y., Morace G., Luberto C., Tomlinson S., Del Poeta M. (2009). App1: An antiphagocytic protein that binds to complement receptors 3 and 2. J. Immunol..

[40] Vogel R.A. (1969). Primary isolation medium for *Cryptococcus neoformans*. Appl. Microbiol..

[41] Diamond R.D., Bennett J.E. (1973). Growth of *Cryptococcus neoformans* within human macrophages in vitro. Infect. Immun..

[42] Levitz S.M., Nong S.-H., Seetoo K.F., Harrison T.S., Speizer R.A., Simons E.R. (1999). *Cryptococcus neoformans* resides in an acidic phagolysosome of human macrophages. Infect. Immun..

[43] Smith L.M., Dixon E.F., May R.C. (2015). The fungal pathogen *Cryptococcus neoformans* manipulates macrophage phagosome maturation. Cell. Microbiol..

[44] Horwitz M.A., Maxfield F.R. (1984). Legionella pneumophila inhibits acidification of its phagosome in human monocytes. J. Cell Biol..

[45] Kronstad J.W., Hu G., Jung W.H. (2013). An encapsulation of iron homeostasis and virulence in *Cryptococcus neoformans*. Trends Microbiol..

[46] Chayakulkeeree M., Johnston S.A., Oei J.B., Lev S., Williamson P.R., Wilson C.F., Zuo X., Leal A.L., Vainstein M.H., Meyer W. (2011). Sec14 is a specific requirement for secretion of phospholipase B1 and pathogenicity of *Cryptococcus neoformans*. Mol. Microbiol..

[47] Chen S.C., Wright L.C., Santangelo R.T., Muller M., Moran V.R., Kuchel P.W., Sorrell T.C. (1997). Identification of extracellular phospholipase B, lysophospholipase, and acyltransferase produced by *Cryptococcus neoformans*. Infect. Immun..

[48] Chen S.C., Wright L.C., Golding J.C., Sorrell T.C. (2000). Purification and characterization of secretory phospholipase B, lysophospholipase and lysophospholipase/transacylase from a virulent strain of the pathogenic fungus *Cryptococcus neoformans*. Biochem. J..

[49] Evans R.J., Li Z., Hughes W.S., Djordjevic J.T., Nielsen K., May R.C. (2015). Cryptococcal phospholipase B1 is required for intracellular proliferation and control of titan cell morphology during macrophage infection. Infect. Immun..

[50] Santangelo R., Zoellner H., Sorrell T., Wilson C., Donald C., Djordjevic J., Shounan Y., Wright L. (2004). Role of extracellular phospholipases and mononuclear phagocytes in dissemination of cryptococcosis in a murine model. Infect. Immun..

[51] Tucker S.C., Casadevall A. (2002). Replication of *Cryptococcus neoformans* in macrophages is accompanied by phagosomal permeabilization and accumulation of vesicles containing polysaccharide in the cytoplasm. Proc. Natl. Acad. Sci. USA.

[52] Johnston S.A., May R.C. (2010). The human fungal pathogen *Cryptococcus neoformans* escapes macrophages by a phagosome emptying mechanism that is inhibited by Arp2/3 complex-mediated actin polymerisation. PLoS Pathog..

[53] Davis M.J., Eastman A.J., Qiu Y., Gregorka B., Kozel T.R., Osterholzer J.J., Curtis J.L., Swanson J.A., Olszewski M.A. (2015). *Cryptococcus neoformans*-induced macrophage lysosome damage crucially contributes to fungal virulence. J. Immunol..

[54] Zaragoza O., Chrisman C.J., Castelli M.V., Frases S., Cuenca-Estrella M., Rodríguez-Tudela J.L., Casadevall A. (2008). Capsule enlargement in *Cryptococcus neoformans* confers resistance to oxidative stress suggesting a mechanism for intracellular survival. Cell. Microbiol..

[55] Nordenfelt P., Tapper H. (2011). Phagosome dynamics during phagocytosis by neutrophils. J. Leukocyte Biol..

[56] Denham S.T., Verma S., Reynolds R.C., Worne C.L., Daugherty J.M., Lane T.E., Brown J.C.S. (2017). Regulated release of cryptococcal polysaccharide drives virulence and suppresses immune infiltration into the central nervous system. Infect. Immun..

[57] Huffnagle G.B. (1996). Role of cytokines in T cell immunity to a pulmonary *Cryptococcus neoformans* infection. Neurosignals.

[58] Osterholzer J.J., Surana R., Milam J.E., Montano G.T., Chen G.-H., Sonstein J., Curtis J.L., Huffnagle G.B., Toews G.B., Olszewski M.A. (2009). Cryptococcal urease promotes the accumulation of immature dendritic cells and a non-protective T2 immune response within the lung. Am. J. Pathol..

[59] Murdock B.J., Teitz-Tennenbaum S., Chen G.-H., Dils A.J., Malachowski A.N., Curtis J.L., Olszewski M.A., Osterholzer J.J. (2014). Early or late IL-10 blockade enhances TH1 and TH17 effector reponses and promotes fungal clearance in mice with cryptococcal lung infection. J. Immunol..

[60] Hardison S.E., Ravi S., Wozniak K.L., Young M.L., Olszewski M.A., Wormley F.L. (2010). Pulmonary infection with an interferon-γ-producing *Cryptococcus neoformans* strain results in classical macrophage activation and protection. Am. J. Pathol..

[61] Eastman A.J., He X., Qiu Y., Davis M.J., Vedula P., Lyons D.M., Park Y.-D., Hardison S.E., Malachowski A.N., Osterholzer J.J. (2015). Cryptococcal heat shock protein 70 homolog ssa1 contributes to pulmonary expansion of *Cryptococcus neoformans* during the afferent phase of the immune response by promoting macrophage M2 polarization. J. Immunol..

[62] Nicola A.M., Robertson E.J., Albuquerque P., Derengowski L.D.S., Casadevall A. (2011). Nonlytic exocytosis of *Cryptococcus neoformans* from macrophages occurs in vivo and is influenced by phagosomal PH. mBio.

[63] Gilbert A.S., Seoane P.I., Sephton-Clark P., Bojarczuk A., Hotham R., Giurisato E., Sarhan A.R., Hillen A., Velde G.V., Gray N.S. (2017). Vomocytosis of live pathogens from macrophages is regulated by the atypical map kinase ERK5. Sci. Adv..

[64] García-Rodas R., Zaragoza O. (2012). Catch me if you can: Phagocytosis and killing avoidance by *Cryptococcus neoformans*. FEMS Immunol. Med. Microbiol..

[65] Johnston S.A., May R.C. (2013). *Cryptococcus* interactions with macrophages: Evasion and manipulation of the phagosome by a fungal pathogen. Cell. Microbiol..

[66] Rodrigues M.L., Fonseca F.L., Frases S., Casadevall A., Nimrichter L. (2009). The still obscure attributes of cryptococcal glucuronoxylomannan. Med. Mycol..

[67] Barbosa F.M., Fonseca F.L., Holandino C., Alviano C.S., Nimrichter L., Rodrigues M.L. (2006). Glucuronoxylomannan-mediated interaction of *Cryptococcus neoformans* with human alveolar cells results in fungal internalization and host cell damage. Microbes Infect..

[68] Barbosa F.M., Fonseca F.L., Figueiredo R.T., Bozza M.T., Casadevall A., Nimrichter L., Rodrigues M.L. (2007). Binding of glucuronoxylomannan to the CD14 receptor in human A549 alveolar cells induces interleukin-8 production. Clin. Vaccine Immunol..

[69] Teixeira P.A.C., Penha L.L., Mendonça-Previato L., Previato J.O. (2014). Mannoprotein MP84 mediates the adhesion of *Cryptococcus neoformans* to epithelial lung cells. Front. Cell. Infect. Microbiol..

[70] Ganendren R., Carter E., Sorrell T., Widmer F., Wright L. (2006). Phospholipase b activity enhances adhesion of *Cryptococcus neoformans* to a human lung epithelial cell line. Microbes Infect..

[71] Stukes S., Coelho C., Rivera J., Jedlicka A.E., Hajjar K.A., Casadevall A. (2016). The membrane phospholipid binding protein annexin A2 promotes phagocytosis and non-lytic exocytosis of *Cryptococcus neoformans* and impacts survival in fungal infection. J. Immunol..

[72] Na Pombejra S., Salemi M., Phinney B.S., Gelli A. (2017). The metalloprotease, Mpr1, engages annexin A2 to promote the transcytosis of fungal cells across the blood–brain barrier. Front. Cell. Infect. Microbiol..

[73] Fang W., Fa Z.-Z., Xie Q., Wang G.-Z., Yi J., Zhang C., Meng G.-X., Gu J.-L., Liao W.-Q. (2017). Complex roles of annexin A2 in host blood–brain barrier invasion by *Cryptococcus neoformans*. CNS Neurosci. Ther..

[74] Aaron P.A., Jamklang M., Uhrig J.P., Gelli A. (2017). The blood–brain barrier internalises *Cryptococcus neoformans* via the epha2-tyrosine kinase receptor. Cell. Microbiol..

[75] Uhlen M., Zhang C., Lee S., Sjöstedt E., Fagerberg L., Bidkhori G., Benfeitas R., Arif M., Liu Z., Edfors F. (2017). A pathology atlas of the human cancer transcriptome. Science.

[76] Guillot L., Carroll S.F., Homer R., Qureshi S.T. (2008). Enhanced innate immune responsiveness to pulmonary Cryptococcus neoformans infection is associated with resistance to progressive infection. Infect. Immun..

[77] Guillot L., Carroll S.F., Badawy M., Qureshi S.T. (2008). *Cryptococcus neoformans* induces IL-8 secretion and CXCl1 expression by human bronchial epithelial cells. Respir. Res..

[78] Heyen L., Müller U., Siegemund S., Schulze B., Protschka M., Alber G., Piehler D. (2016). Lung epithelium is the major source of IL-33 and is regulated by IL-33-dependent and IL-33-independent mechanisms in pulmonary cryptococcosis. Pathog. Dis..

[79] Chang Y.C., Cherniak R., Kozel T.R., Granger D.L., Morris L.C., Weinhold L.C., Kwon-Chung K.J. (1997). Structure and biological activities of acapsular *Cryptococcus neoformans* 602 complemented with the cap64 gene. Infect. Immun..

[80] Chang Y.C., Kwon-Chung K.J. (1998). Isolation of the third capsule-associated gene, cap60, required for virulence in *Cryptococcus neoformans*. Infect. Immun..

[81] Mahajan K.R., Roberts A.L., Curtis M.T., Fortuna D., Dharia R., Sheehan L. (2016). Diagnostic challenges of *Cryptococcus neoformans* in an immunocompetent individual masquerading as chronic hydrocephalus. Case Rep. Neurol. Med..

[82] Cruickshank J.G., Cavill R., Jelbert M. (1973). *Cryptococcus neoformans* of unusual morphology. Appl. Microbiol..

[83] Feldmesser M., Kress Y., Casadevall A. (2001). Dynamic changes in the morphology of *Cryptococcus neoformans* during murine pulmonary infection. Microbiology.

[84] Zaragoza O., García-Rodas R., Nosanchuk J.D., Cuenca-Estrella M., Rodríguez-Tudela J.L., Casadevall A. (2010). Fungal cell gigantism during mammalian infection. PLoS Pathog..

[85] Okagaki L.H., Strain A.K., Nielsen J.N., Charlier C., Baltes N.J., Chrétien F., Heitman J., Dromer F., Nielsen K. (2010). Cryptococcal cell morphology affects host cell interactions and pathogenicity. PLoS Pathog..

[86] Wang J.-M., Zhou Q., Cai H.-R., Zhuang Y., Zhang Y.-F., Xin X.-Y., Meng F.-Q., Wang Y.-P. (2014). Clinicopathological features of pulmonary cryptococcosis with cryptococcal titan cells: A comparative analysis of 27 cases. Int. J. Clin. Exp. Pathol..

[87] Chrisman C.J., Albuquerque P., Guimaraes A.J., Nieves E., Casadevall A. (2011). Phospholipids trigger *Cryptococcus neoformans* capsular enlargement during interactions with amoebae and macrophages. PLoS Pathog..

[88] Okagaki L.H., Wang Y., Ballou E.R., O’Meara T.R., Bahn Y.-S., Alspaugh J.A., Xue C., Nielsen K. (2011). Cryptococcal titan cell formation is regulated by G-protein signaling in response to multiple stimuli. Eukaryot. Cell.

[89] Crabtree J.N., Okagaki L.H., Wiesner D.L., Strain A.K., Nielsen J.N., Nielsen K. (2012). Titan cell production enhances the virulence of *Cryptococcus neoformans*. Infect. Immun..

[90] O’Meara T.R., Holmer S.M., Selvig K., Dietrich F., Alspaugh J.A. (2013). *Cryptococcus neoformans* rim101 is associated with cell wall remodeling and evasion of the host immune responses. mBio.

[91] Ost K.S., O’Meara T.R., Huda N., Esher S.K., Alspaugh J.A. (2015). The *Cryptococcus neoformans* alkaline response pathway: Identification of a novel rim pathway activator. PLoS Genet..

[92] Gerstein A.C., Fu M.S., Mukaremera L., Li Z., Ormerod K.L., Fraser J.A., Berman J., Nielsen K. (2015). Polyploid titan cells produce haploid and aneuploid progeny to promote stress adaptation. mBio.

[93] Okagaki L.H., Nielsen K. (2012). Titan cells confer protection from phagocytosis in *Cryptococcus neoformans* infections. Eukaryot. Cell.

[94] García-Barbazán I., Trevijano-Contador N., Rueda C., de Andrés B., Pérez-Tavárez R., Herrero-Fernández I., Gaspar M.L., Zaragoza O. (2016). The formation of titan cells in *Cryptococcus neoformans* depends on the mouse strain and correlates with induction of Th2-type responses. Cell. Microbiol..

[95] Wiesner D.L., Specht C.A., Lee C.K., Smith K.D., Mukaremera L., Lee S.T., Lee C.G., Elias J.A., Nielsen J.N., Boulware D.R. (2015). Chitin recognition via chitotriosidase promotes pathologic type-2 helper T cell responses to cryptococcal infection. PLoS Pathog..

[96] Xie S., Sao R., Braun A., Bottone E.J. (2012). Difference in *Cryptococcus neoformans* cellular and capsule size in sequential pulmonary and meningeal infection: A postmortem study. Diagn. Microbiol. Infect. Dis..

[97] Charlier C., Chrétien F., Baudrimont M., Mordelet E., Lortholary O., Dromer F. (2005). Capsule structure changes associated with *Cryptococcus neoformans* crossing of the blood–brain barrier. Am. J. Pathol..

[98] Rivera J., Feldmesser M., Cammer M., Casadevall A. (1998). Organ-dependent variation of capsule thickness in *Cryptococcus neoformans* during experimental murine infection. Infect. Immun..

[99] Bouklas T., Fries B.C. (2015). Aging as an emergent factor that contributes to phenotypic variation in *Cryptococcus neoformans*. Fungal Genet. Biol. FG B.

[100] Powell C.D., Quain D.E., Smart K.A. (2003). Chitin scar breaks in aged saccharomyces cerevisiae. Microbiology.

[101] Bouklas T., Pechuan X., Goldman D.L., Edelman B., Bergman A., Fries B.C. (2013). Old *Cryptococcus neoformans* cells contribute to virulence in chronic cryptococcosis. mBio.

[102] Jain N., Cook E., Xess I., Hasan F., Fries D., Fries B.C. (2009). Isolation and characterization of senescent *Cryptococcus neoformans* and implications for phenotypic switching and pathogenesis in chronic cryptococcosis. Eukaryot. Cell.

[103] García-Rodas R., Cordero R.J.B., Trevijano-Contador N., Janbon G., Moyrand F., Casadevall A., Zaragoza O. (2014). Capsule growth in *Cryptococcus neoformans* is coordinated with cell cycle progression. mBio.

[104] Bouklas T., Jain N., Fries B.C. (2017). Modulation of replicative lifespan in *Cryptococcus neoformans*: Implications for virulence. Front. Microbiol..

[105] Coelho C., Casadevall A. (2016). Cryptococcal therapies and drug targets: The old, the new and the promising. Cell. Microbiol..

[106] Eisenman H.C., Casadevall A. (2012). Synthesis and assembly of fungal melanin. Appl. Microbiol. Biotechnol..

[107] Liu G.Y., Nizet V. (2009). Color me bad: Microbial pigments as virulence factors. Trends Microbiol..

[108] Pukkila-Worley R., Gerrald Q.D., Kraus P.R., Boily M.-J., Davis M.J., Giles S.S., Cox G.M., Heitman J., Alspaugh J.A. (2005). Transcriptional network of multiple capsule and melanin genes governed by the *Cryptococcus neoformans* cyclic amp cascade. Eukaryot. Cell.

[109] Noverr M.C., Williamson P.R., Fajardo R.S., Huffnagle G.B. (2004). CNLAC1 is required for extrapulmonary dissemination of *Cryptococcus neoformans* but not pulmonary persistence. Infect. Immun..

[110] Salas S.D., Bennett J.E., Kwon-Chung K.J., Perfect J.R., Williamson P.R. (1996). Effect of the laccase gene *CNLAC1*, on virulence of *Cryptococcus neoformans*. J. Exp. Med..

[111] Sabiiti W., Robertson E., Beale M.A., Johnston S.A., Brouwer A.E., Loyse A., Jarvis J.N., Gilbert A.S., Fisher M.C., Harrison T.S. (2014). Efficient phagocytosis and laccase activity affect the outcome of HIV-associated cryptococcosis. J. Clin. Investig..

[112] Lev S., Li C., Desmarini D., Saiardi A., Fewings N.L., Schibeci S.D., Sharma R., Sorrell T.C., Djordjevic J.T. (2015). Fungal inositol pyrophosphate IP_7_ is crucial for metabolic adaptation to the host environment and pathogenicity. mBio.

[113] Li C., Lev S., Desmarini D., Kaufman-Francis K., Saiardi A., Silva A.P.G., Mackay J.P., Thompson P.E., Sorrell T.C., Djordjevic J.T. (2017). Ip3-4 kinase Arg1 regulates cell wall homeostasis and surface architecture to promote clearance of *Cryptococcus neoformans* infection in a mouse model. Virulence.

[114] Li C., Lev S., Saiardi A., Desmarini D., Sorrell T.C., Djordjevic J.T. (2016). Identification of a major IP_5_ kinase in *Cryptococcus neoformans* confirms that PP-IP_5_/IP_7_, not IP_6_, is essential for virulence. Sci. Rep..

[115] Lev S., Kaufman-Francis K., Desmarini D., Juillard P.G., Li C., Stifter S.A., Feng C.G., Sorrell T.C., Grau G.E.R., Bahn Y.-S. (2017). Pho4 is essential for dissemination of *Cryptococcus neoformans* to the host brain by promoting phosphate uptake and growth at alkaline PH. mSphere.

[116] Toh-e A., Ohkusu M., Li H.-M., Shimizu K., Takahashi-Nakaguchi A., Gonoi T., Kawamoto S., Kanesaki Y., Yoshikawa H., Nishizawa M. (2015). Identification of genes involved in the phosphate metabolism in *Cryptococcus neoformans*. Fungal Genet. Biol..

[117] O’Meara T.R., Norton D., Price M.S., Hay C., Clements M.F., Nichols C.B., Alspaugh J.A. (2010). Interaction of *Cryptococcus neoformans* rim101 and protein kinase a regulates capsule. PLoS Pathog..

[118] Ost K.S., Esher S.K., Leopold Wager C.M., Walker L., Wagener J., Munro C., Wormley F.L., Alspaugh J.A. (2017). Rim pathway-mediated alterations in the fungal cell wall influence immune recognition and inflammation. mBio.

[119] Singh A., MacKenzie A., Girnun G., Del Poeta M. (2017). Analysis of sphingolipids, sterols, and phospholipids in human pathogenic *Cryptococcus* strains. J. Lipid Res..

[120] McQuiston T., Luberto C., Del Poeta M. (2011). Role of sphingosine-1-phosphate (S1P) and S1P receptor 2 in the phagocytosis of *Cryptococcus neoformans* by alveolar macrophages. Microbiology.

[121] McQuiston T., Luberto C., Del Poeta M. (2010). Role of host sphingosine kinase 1 in the lung response against cryptococcosis. Infect. Immun..

[122] Shea J.M., Kechichian T.B., Luberto C., Del Poeta M. (2006). The cryptococcal enzyme inositol phosphosphingolipid-phospholipase c confers resistance to the antifungal effects of macrophages and promotes fungal dissemination to the central nervous system. Infect. Immun..

[123] Farnoud A.M., Bryan A.M., Kechichian T., Luberto C., Del Poeta M. (2015). The granuloma response controlling cryptococcosis in mice depends on the sphingosine kinase 1–sphingosine 1-phosphate pathway. Infect. Immun..

[124] Rittershaus P.C., Kechichian T.B., Allegood J.C., Merrill A.H., Hennig M., Luberto C., Del Poeta M. (2006). Glucosylceramide synthase is an essential regulator of pathogenicity of *Cryptococcus neoformans*. J. Clin. Investig..

[125] Singh A., Wang H., Silva L.C., Na C., Prieto M., Futerman A.H., Luberto C., Del Poeta M. (2012). Methylation of glycosylated sphingolipid modulates membrane lipid topography and pathogenicity of *Cryptococcus neoformans*. Cell. Microbiol..

[126] Raj S., Nazemidashtarjandi S., Kim J., Joffe L., Zhang X., Singh A., Mor V., Desmarini D., Djordjevic J., Raleigh D.P. (2017). Changes in glucosylceramide structure affect virulence and membrane biophysical properties of *Cryptococcus neoformans*. Biochimica et Biophysica Acta (BBA) Biomembranes.

[127] Farnoud A.M., Mor V., Singh A., Del Poeta M. (2014). Inositol phosphosphingolipid phospholipase c1 regulates plasma membrane atpase (pma1) stability in *Cryptococcus neoformans*. FEBS Lett..

[128] Beattie S.R., Mark K.M.K., Thammahong A., Ries L.N.A., Dhingra S., Caffrey-Carr A.K., Cheng C., Black C.C., Bowyer P., Bromley M.J. (2017). Filamentous fungal carbon catabolite repression supports metabolic plasticity and stress responses essential for disease progression. PLoS Pathog..

[129] Pirofski L.-A., Casadevall A. (2017). Immune-mediated damage completes the parabola: *Cryptococcus neoformans* pathogenesis can reflect the outcome of a weak or strong immune response. mBio.

[130] Janbon G., Ormerod K.L., Paulet D., Byrnes E.J., Yadav V., Chatterjee G., Mullapudi N., Hon C.-C., Billmyre R.B., Brunel F. (2014). Analysis of the genome and transcriptome of *Cryptococcus neoformans* var. Grubii reveals complex rna expression and microevolution leading to virulence attenuation. PLoS Genet..

[131] Dufaud C., Rivera J., Rohatgi S., Pirofski L.-A. (2018). Naïve B cells reduce fungal dissemination in *Cryptococcus neoformans* infected Rag1^−/−^ mice. Virulence.

[132] Rohatgi S., Nakouzi A., Carreño L.J., Slosar-Cheah M., Kuniholm M.H., Wang T., Pappas P.G., Pirofski L.-A. (2018). Antibody and B cell subset perturbations in human immunodeficiency virus-uninfected patients with cryptococcosis. Open Forum Infect. Dis..

[133] Wiesner D.L., Smith K.D., Kashem S.W., Bohjanen P.R., Nielsen K. (2017). Different lymphocyte populations direct dichotomous eosinophil or neutrophil responses to pulmonary *Cryptococcus* infection. J. Immunol..

